# *N*‑Alkylamino-Functionalized Multiwalled Carbon Nanotubes for Advanced Grease Applications: Stability and Tribological Enhancement

**DOI:** 10.1021/acsomega.5c07880

**Published:** 2025-10-28

**Authors:** Ilona Scudło, Julia Woch, Szymon Ruczka, Kamil Korasiak, Ewa Sabura, Katarzyna Gębura, Agata Blacha-Grzechnik, Sławomir Boncel

**Affiliations:** 1 Faculty of Chemistry, Department of Organic Chemistry, Bioorganic Chemistry and Biotechnology, NanoCarbon Group, 49569 Silesian University of Technology, 4 Bolesława Krzywoustego Street, Gliwice 44-100, Poland; 2 Łukasiewicz Research Network, Institute of Heavy Organic Synthesis ‘Blachownia’, 9 Energetyków Street, Kędzierzyn-Koźle 47-225, Poland; 3 Faculty of Chemistry, Department of Physical Chemistry and Technology of Polymers, 49569 Silesian University of Technology, 9 Ks. Marcina Strzody Street, Gliwice 44-100, Poland; 4 Centre for Organic and Nanohybrid Electronics (CONE), 49569 Silesian University of Technology, 22B Stanisława Konarskiego Street, Gliwice 44-100, Poland; 5 NanoCarbon Group Ltd., 7 Ks. Marcina Strzody Street, Gliwice 44-100, Poland

## Abstract

Advanced lubricants play a critical role in modern industry by reducing friction, minimizing wear, and extending the service life of heavily loaded mechanical systems. Addressing these challenges requires the development of nanocomponents capable of simultaneously improving multiple tribological parameters without compromising the consistency or manufacturability. This study investigates the potential of *N*-alkylamino functionalized multiwalled carbon nanotubes (MWCNTs) as high-performance nanoadditives for industrial grease formulations. The aim is to evaluate their impact on the dispersion stability and key tribological properties under severe operating conditions. *N*-alkylamino MWCNTs were synthesized via one-step nucleophilic addition of terminal *n*-alkylamines with chain lengths of C_6_, C_12_, and C_16_ to pristine MWCNTs yielding the functionalization degrees equal to 3.2, 2.8, and 10.3 wt %, respectively. This approach ensured atom economy, eliminated side products and problematic wastes, and operated under relatively mild conditions with high overall efficiency. The resulting nanoadditives were dispersed in a base mineral oil and incorporated at as low as 0.02 wt % into lithium-based greases containing nanographite and MoS_2_ as the typical commercial additives. The functionalized MWCNTs demonstrated excellent time and operational stability in the base oil, with no observable sedimentation over 7 days. Greases formulated with *N*-alkylamino-functionalized MWCNTs demonstrated markedly superior tribological performance, achieving a weld load of up to 490 kG (versus 250 kG for the commercial reference), reduced wear scar diameters of 0.49–0.50 mm (approximately 11% lower), and an exceptionally low SRV friction coefficient of 0.1030, representing a reduction of up to 84% compared to the commercial grease. Notably, these enhancements were achieved while maintaining an NLGI grease consistency class of 2, ensuring practical applicability and processability. This approach offers a scalable route to next-generation lubricants suitable for highly loaded tribo–pairs, precision mechanical assemblies, and energy-efficient drivetrains.

## Graphical abstract

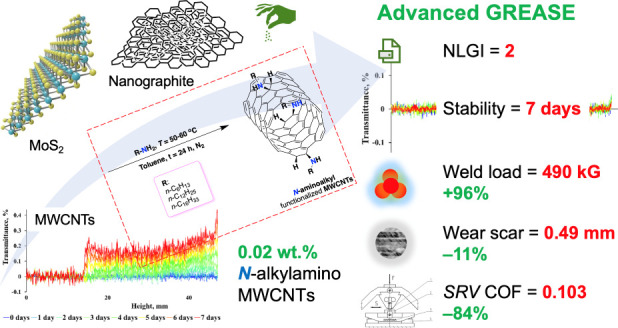

## Introduction

Carbon nanotubes (CNTs), composed of a lubricious *C*-sp^2^-hybridized carbon framework, exhibit outstanding mechanical strength and hold considerable promise for enhancing the tribological performance of greases, particularly in high-viscosity lubrication regimes.^1–5^ At the same time, one of the key challenges in developing high-performance CNT-based lubricants lies in formulating stable CNT-grease systems that effectively suppress or delay nanotube reagglomeration. Consequently, efforts to enhance the performance of plastic greases are increasingly centered on the design of morphologically and physicochemically tailored CNTs—particularly functionalized CNT variants capable of stabilizing grease formulations through steric hindrance mechanisms.^6^

Greases, widely employed as industrial and automotive lubricants, are typically composed of four key components: (a) a base oil, (b) a thickening agent, (c) performance-enhancing (nano)­additives to mitigate wear and seizure, and (d) corrosion inhibitors. Specifically, serving as the lubricant primary fluid component, base oils typically constitute 75–90 wt % of the grease. Base oils are generally classified into three categories: (1) mineral oils, derived from refined crude oil and favored for their cost-effectiveness under moderate operating conditions; (2) synthetic oils, engineered for enhanced thermal and oxidative stability under extreme temperatures and pressures; and (3) biodegradable oils, sourced from renewable materials such as vegetable oils, offering environmentally sustainable lubrication solutions. In turn, thickeners impart the semisolid, 3D-structure characteristic of greases by entrapping the base oil within their matrix, thereby defining the grease consistency classification. Typically comprising 5–15 wt % of the formulation, thickeners include metallic soaps such as lithium-, calcium-, and aluminum-based systems. Lithium soaps are especially favored for their balanced performance and broad compatibility. Nonsoap alternatives, including clay, polyurea, and silica, are valued for their superior thermal stability and resistance to water washout. Functional (nano)­additives are incorporated into grease formulations to achieve targeted performance characteristics, tailoring the lubricant to specific operational demands. Typically comprising 1–10 wt % of the total composition, these additives include antiwear agents, which form protective films on metal surfaces to mitigate wear under high loads; extreme pressure additives, which prevent metal-to-metal contact under severe loading, thereby reducing surface damage; oxidation inhibitors, which enhance thermal stability and prolong grease life by suppressing degradation from heat and oxygen; rust and corrosion inhibitors, which safeguard metal components in moisture-prone environments; and tackifiers, which improve adhesion and retention, ensuring the grease remains in place even on vertical or high-vibration surfaces.^7^ Understanding the roles and interactions of these components is essential for formulating greases that meet the demanding requirements of various industrial and automotive applications.

CNTs exhibit strong potential as efficient and cost-effective alternatives to the conventional functional additives in lubricant formulations.^8–10^ At present, nanotechnology-based products facilitate the formation of a protective layer at the interface of metal surfaces, filling in microscopic irregularities and providing a smoother surface. There are two main groups of mechanisms through which nanoparticles have a beneficial effect on lubrication. The first mechanism is the direct effect on improving the properties of the oil film, which leads to a reduction in friction through the formation of a protective film and a reduction in friction through the ball-bearing effect or rolling effect.^1^ The second group of mechanisms consists of repair mechanisms that affect the structure of friction surfaces through the filling effect and/or the polishing effect.^11^ The research primarily targets lubrication interfaces involving bearing steels—specialized alloys used in the fabrication of rolling elements such as shafts, rings, balls, and needles. Bearing components are characterized by a high carbon content (ca. 1%). The use of carbon nanomaterials, in particular CNTs and graphene as modern additives, can potentially have a positive effect on lubricating properties by improving the lubricating mechanisms at the metal interface.^12^

A primary limitation of multiwalled CNTs (MWCNTs)—a synthetically less demanding and more cost-effective form of CNTs—is their poor dispersibility in conventional base oils. Pristine MWCNTs tend to aggregate rapidly in liquid media, resulting in a swift sedimentation. Nonetheless, the inherent chemical structure of MWCNTs permits diverse functionalization strategies that can significantly enhance their dispersion stability in base oils. Ultimately, the careful selection of functionalizing agents—both noncovalent and covalent—can significantly improve the tribological performance of lubricants incorporating modified CNTs at the friction interface. Chemical modification of MWCNTs predominantly involves the application of surfactants,^13–15^ which enhance the nanomaterial wettability^16^ and mitigate their propensity to agglomerate in dispersions. In turn, physical modifications primarily encompass controlled grinding and ultrasonication processes^17,18^ that can induce substantial structural alterations, including the exfoliation of MWCNTs into graphene-like sheets. These treatments may also influence the dimensional distribution (length and diameter) of MWCNTs as well as modify their surface physicochemical properties.^19,20^ Physical modification of CNTs alone is often insufficient to achieve stable dispersions in oil-based media. Therefore, challenges associated with nanoparticle agglomeration have necessitated chemical functionalization strategies. Indeed, properly tailored chemical modifications emerge as enhancing the compatibility of CNTs with the continuous phase, facilitating dispersion stabilization through steric or electrostatic mechanisms. It was demonstrated that the surface chemical modifications of MWCNTs had a beneficial effect on the interaction mechanisms in the tribo-pairs. In addition to forming a lubricating film, they facilitate the sliding of the two friction surfaces, smooth out irregularities on the contact surface of the working metals, and improve load transfer at the friction nodes. Nevertheless, a critical challenge remains in achieving stable dispersions of MWCNTs within hydrocarbons, which constitute the fundamental base components of most lubricants. For instance, surface amination of CNTs significantly improved their compatibility with lubricating matrices, promoting a more uniform dispersion within the continuous phase of the lubricant formulation. This treatment enhanced distribution effectively reduces friction and wear at sliding interfaces.^21^ Furthermore, amino-functionalized CNTs facilitate their incorporation into lubricating systems and promote the formation of protective tribofilms on contact surfaces. Acting akin to solid lubricants, these layers effectively reduce direct asperity contact, thereby minimizing friction and wear.^22^ Amino functional groups on CNTs also can interact synergistically with diverse lubricant additives, altering the rheological behavior of the formulation and enhancing the overall tribological performance. Such interactions promote the development of more stable and efficient lubrication regimes.^23^ For instance, the amino-functionalizations were applied to graphene oxide (GO) and hexagonal boron nitride nanoplatelets (h-BNNPs).^24^ Likewise, GO and fullerene C_60_ functionalized with diamines— including 1,6-hexanediamine, 1,8-octanediamine, 1,10-decanediamine, and 1,12-dodecanediamine— have exhibited markedly enhanced antiwear properties when incorporated as lubricant additives.^25^ The study demonstrated that the nanocomposite materials significantly lowered friction coefficients and wear cross-sectional areas, with the composite modified by 1,12-dodecanediamine-functionalized GO and C_60_ achieving up to a 30% reduction in friction and a 98% decrease in wear under conditions of high contact stress.

In this study, we report—for the first time—the functionalization of MWCNTs using *n*-hexylamine, *n*-dodecylamine, and *n*-hexadecylamine to develop a series of plastic greases tailored for high-load tribological contacts. Our approach addresses a critical gap in the field: *N*-alkylamino functionalization of MWCNTs has not been studied to date, neither as a standalone modification nor in the context of grease formulations, and its impact on tribological performance at ultralow additive concentrations (0.02 wt %) remains entirely unexplored. The target greases are specifically designed for lubrication of critical high-load interfaces predominantly composed of bearing steel used in rolling element components. Specifically, the formulated *N*-alkylamino MWCNT greases were found as ideally suited for lubrication points in machinery and equipment across metallurgy, automotive, engineering, steel, and mining sectors. Comprehensive characterization revealed that the novel greases significantly outperformed existing commercial formulations, demonstrating superior weld load capacity, reduced wear scar diameter, and lower friction coefficients as the key functional metrics.

## Materials and Methods

### Chemicals and Materials

The study utilized unmodified commercially available Nanocyl NC7000 MWCNTs (length, ca. 1.5 μm; outer diameter, ca. 9.5 nm), produced by catalytic chemical vapor deposition (Nanocyl Ltd., Sambreville, Belgium). Commercially available aliphatic amines (Acros Organics, Belgium): *n*-hexylamine (C6) [CAS 11–26–2] (>99 wt %), *n*-dodecylamine (C12) [CAS 124–22–1] (>98 wt %), and *n*-hexadecylamine (C16) CAS 143–27–1 (>90 wt %) were used as purchased.

### *N*-Alkylamino Functionalization of CNTs

The optimized *N-*alkylamino functionalization of MWCNTs was carried out with aliphatic amines with different chain lengths. The selection of the functionalization unit was dictated by the goal of improving the antiwear and antifriction properties of the medium, in which the dispersed nanomaterial could form more stable dispersions than those containing in unmodified/pristine MWCNTs. The three target products were MWCNT-NH-*n*-hexylamine (MWCNT-NH-C6), MWCNT-NH-*n*-dodecylamine (MWCNT-NH-C12), and MWCNT-NH-*n*-hexadecylamine (MWCNT-NH-C16), obtained by the treatment of MWCNTs with *n*-hexylamine, *n*-dodecylamine, and *n*-hexadecylamine, respectively. The functionalization of MWCNTs was carried out according to the following procedure. Briefly, as-purchased MWCNTs (0.5020 g) were predried in a laboratory dryer for 2 h at 120 °C, yielding dried MWCNTs (0.5000 g). The dried MWCNTs were treated with an excess of the appropriate amine (10.00 g) in toluene (100 mL) as the solvent of choice. The mixture was subjected to ultrasonication using a Sonics VC 505 homogenizer under the following conditions: *t* = 1 h, amplitude 70%, operation 7/3 s, *T* = 40 °C, with a constant cooling of the system by thermostatting. The resulting suspension was stirred for 24 h under a nitrogen atmosphere at 50–60 °C, and then it was diluted with *n*-hexane and filtered under vacuum through a Pabiantex PPT 2708 polypropylene filter cloth. The raw product was washed with *n*-hexane until the excess amine was completely removed. Qualitative determination of the free, unreacted primary amine was carried out by titration method using a Tashir reagent. The obtained derivative was then dried in a laboratory dryer for 2–4 h at 110–115 °C until it reached a constant weight equal to 0.5140, 0.5115, and 0.5451 g for C6-, C12-, and C16-amine treatment, respectively.

### Characterization of the *N-*alkylamino-Functionalized MWCNTs

Cryogenic transmission electron microscopy (cryo-TEM) images were obtained using a Tecnai F20 X TWIN microscope (FEI Company, Hillsboro, Oregon, USA) equipped with a field emission gun operating at an acceleration voltage of 200 kV. The images were recorded on a Gatan Rio 16 CMOS 4k camera (Gatan Inc., Pleasanton, California, USA) and processed with Gatan Microscopy Suite (GMS) software (Gatan Inc., Pleasanton, California, USA). Specimen preparation was done by vitrification of the aqueous solutions on grids with holey carbon film (Quantifoil R 2/2; Quantifoil Micro Tools GmbH, Großlöbichau, Germany). Prior to use, the grids were activated for 15 s in oxygen plasma using a Femto plasma cleaner (Diener Electronic, Ebhausen, Germany). Cryo-samples were prepared by applying a droplet (3 μL) of the suspension to the grid, blotting with filter paper, and immediately freezing in liquid ethane using a fully automated blotting device, Vitrobot Mark IV (Thermo Fisher Scientific, Waltham, Massachusetts, USA). After preparation, the vitrified specimens were kept under liquid nitrogen until they were inserted into a cryo-TEM-holder Gatan 626 (Gatan Inc., Pleasanton, USA) and analyzed in the TEM at – 178 °C. The Mettler Toledo TGA 2 Thermobalance with the STAR^e^ Thermal Analysis Software was used. The samples (ca. 10 mg) were heated in an open platinum crucible (Pt 70 μL), in the temperature range from 30 to 800 °C at the heating rate of 20 °C min^–1^, in the dynamic (100 mL min^–1^) nitrogen atmosphere. Raman spectra were acquired with an inVia Confocal Raman microscope (Renishaw, New Mills, United Kingdom) with an excitation source of monochromatic red light at 633 nm with a resolution higher than 1.5 cm^–1^. To focus light beam, a ×20 objective of an Olympus optical microscope was used. With a CCD detector camera, the spectra were collected from 100 to 3500 cm^–1^ over 10 s (three accumulations). Raman spectra were deconvoluted based on literature reports^26^ with Fityk software^27^ using Lorentzian and Gaussian peak shape. Fourier transform infrared spectroscopy (FTIR) was used to confirm the formation of the expected chemical (amine) bonds after the modification of MWCNTs. The chemical structures of different nanomaterials products with aliphatic amines were characterized using a Nicolet 6700 FTIR spectrophotometer (KBr powder, NaCl film, and ATR FTIR using ZnSe 60° crystal) over a range of 4000–400 cm^–1^ with a resolution of 4 cm^–1^. FTIR spectra were collected for 16 scans in transmission mode using KBr powder and NaCl film and for 32 scans in attenuated total reflectance (SMART ARK ATR). Solids (MWCNTs, functionalized MWCNTs, *n*-dodecylamine, and *n*-hexadecylamine) were analyzed using a pellet of 1 mg of the test sample per 350 mg of KBr, while the only liquid (*n*-hexylamine) was analyzed as a film on NaCl. Concerning X-ray photoelectron spectroscopy (XPS), powder samples were pressed using clean borosilicate glass onto a strip of electroconductive copper tape affixed to an indium–tin-oxide (ITO)-coated glass substrate, effectively minimizing charging effects during analysis. All sample preparation was conducted under ambient atmospheric conditions. XPS measurements were performed in an ultrahigh vacuum environment (base pressure: 3×10^–9^ Torr) using a Kratos Axis Supr+ spectrometer equipped with a monochromatic Al Kα X-ray source (10 mA, 15 kV). Survey spectra were acquired with a pass energy (PE) of 160 eV and a step size of 0.9 eV. For detailed chemical analysis, high-resolution scans were conducted with a PE of 20 eV and an energy step of 0.05 eV. The dwell time was set to 0.2 s per step, with signal accumulation over 10 iterations for the C 1s and O 1s regions and 15 iterations for the N 1s region to enhance detection accuracy. The binding energy (BE) scale of the analyzer was calibrated against the Au 4f_7/2_ peak (84.0 eV) of a gold-coated reference sample mounted on the same sample stage.^28^

### Base Oil

The base oil used in the application part was SN650 mineral oil [CAS: 64742-54-7], which belongs to the Group I base oils according to the API (American Petroleum Institute) classification.

### Nanotube Dispersions in the Base Oil

Dispersions in the base oil were prepared from pristine MWCNTs and their *N*-alkylamino derivatives. Table S1 shows the scheme of obtaining dispersions of MWNCTs and MWCNT derivatives in the lubricant oil base SN 650.

### Spectroscopic Evaluation of the Dispersion Stability by Measuring Transmittance Changes over Time

The stability of the nanotube formulations was assessed using the multiple light scattering (MLS) method using a Turbiscan Thermo instrument equipped with a laser of an 880 nm wavelength. While the beam illuminated the sample, the light was scattered by the dispersed particles and recorded by the detector at an angle of 45°. The detector collected data every 40 μm, depending on the height of the sample in the measuring vessel. The backscattered light intensity (RW) and transmittance (*T*) were measured as a function of the height of the liquid layer in the measuring vessel. According to the theory of light scattering (Mie theory), the value of RW depends on the size and concentration of particles in the dispersion:

$$\mathrm{RW}=\frac{1}{\sqrt{l^{*}}}$$

$$l^{*}\left(\varphi ,d\right)=\frac{2d}{3\varphi \left(1-g\right)Q_{s}}$$

where l* is average photon path in the dispersed system, φ is volume fraction of particles, *d* is average particle diameter, and *g* and *Q*_*s*_ are optical parameters determined from the light scattering theory proposed by Mie. Changes in RW and *T*, depending on the height of the sample in the measuring vessel, reflect changes in the microstructure of the system—migration of dispersed phase particles or changes in their size, such as agglomeration or sedimentation. The TSI (Turbiscan Stability Index), determined by quantitatively comparing all scans in a sample, was used to compare the stability over time of the individual samples:

$$\mathrm{TSI}=\sqrt{\frac{\sum_{i=1}^{n}{\left(x_{i}-x_{\mathrm{RW}}\right)}^{2}}{n-1}}$$

where *x*_*i*_ = the average scattered light intensity at a given time, *x*_*RW*_ = the average *x*_*i*_, and *n* is the total number of scans. The lower the TSI value, the less changes occur in the sample.^29^

### Composition of the Plastic Greases

The plastic greases for highly loaded tribo-pairs contained (a) base grease, obtained from refined mineral oil (kinematic viscosity of 140 to 160 mm^2^ s^–1^ at 40 °C) enriched with a thickener in the form of lithium soap of 12-hydroxystearic acid and (b) liquid and solid lubricating (nano)­additives. As liquid lubricating additives, they contained 0.5 wt % of a mixture of primary and secondary zinc dialkyldithiophosphates, available under the trade name HiTEC 1656, while as solid lubricating additives, they contained 0.25 wt % of nanographite (specific surface area 250–400 m^2^ g^–1^, a lateral size 100–500 nm, thickness 10–300 nm), 0.5 wt % MoS_2_ (particle size in the range of 100–2000 nm), and 0.02 wt % of variously *N*-alkylamino functionalized MWCNTs. The share of the thickener in the base lubricant was 1 and 4 wt %. A mixture of primary and secondary zinc dialkyldithiophosphates acts as an inhibitor of bearing wear, oxidation, and corrosion. The commercially available HiTEC 1656 product of this category contained ≥75 wt % zinc dialkyldithiophosphate [CAS 85940-28-9] as well as heavy petroleum distillates: 15–25 wt % [CAS 64742-54-7] and 0.5–10 wt % [CAS 64742-65].

### Manufacturing the Greases

The plastic greases were prepared according to generally known procedures by homogenizing mineral oil with 12-hydroxystearic acid lithium soap at 5 atm at 230 °C; the consistency according to NLGI (National Lubricating Grease Institute) was from 0 to 1. Then, all lubricating additives were added and the mixture was homogenized at 5 atm and 25 °C using PandaPLUS 2000, GEA (Germany) at 0.3–5 kg h^–1^ depending on a sample weight.^30^

### Determination of Tribological Parameters

*N*-alkylamino MWCNTs were evaluated as antiseizure and antiwear nanoadditives in the reference and four model plastic greases (1–4). Key tribological performance metrics, including weld load, wear scar diameter, and SRV friction coefficient, were assessed using an SRV oscillating tribometer [German: Schwingung (oscillation/vibration), Reibung (friction), Verschleiß (wear)] (SRV/Schwingung Reibwert Prüfgerät). The results were benchmarked against a commercial grease to elucidate the influence of the new functionalized CNTs on enhancing antiseizure and antiwear properties. The consistency of the lubricating greases was evaluated in accordance with the National Lubricating Grease Institute (NLGI) classification, spanning grades from 000 to 5, as specified by the PN-85/C-04095 standard. Notably, the incorporation of the investigated additives did not alter the consistency class, which remained stable at NLGI grade 2 throughout all formulations. The weld load value for plastic greases was determined according to the methodology in PN-EN ISO 20623:2018–02 “Petroleum products and related products—determination of antiseizure and antiwear properties of lubricants—four-ball method”. The oscillatory friction coefficient SRV was determined according to the methodology described in ASTM D5707 “Standard test method for measuring friction and wear properties of lubricating grease using a high-frequency, linear-oscillation (SRV) test machine”. This testing method can be used to determine the wear properties and coefficient of friction of lubricating greases at selected temperatures and loads specified for use in applications where high-speed vibrational or start–stop motions are present for extended periods of time under initial high Hertzian-point contact pressures. This test method covers a procedure for determining a lubricating grease coefficient of friction and its ability to protect against wear when subjected to high-frequency, linear-oscillation motion using an SRV test machine at a test load of 200 N, frequency of 50 Hz, stroke amplitude of 1.00 mm, duration of 2 h, and temperature within the range of the test machine from ambient to 280 °C. The scar diameter was determined in accordance with the methodology also contained in PN-EN ISO 20623:2018–02. This international standard was developed in accordance with internationally recognized principles on standardization established in the Decision on Principles for the Development of International Standards, Guides, and Recommendations issued by the World Trade Organization Technical Barriers to Trade (TBT) Committee.

## Results and Discussion

*N-*alkylaminoation was employed as a one-step covalent functionalization strategy to modify the structure of MWCNTs (Figure 1), facilitating the formation of stable dispersions in hydrocarbon-based oils.

**Figure 1:**
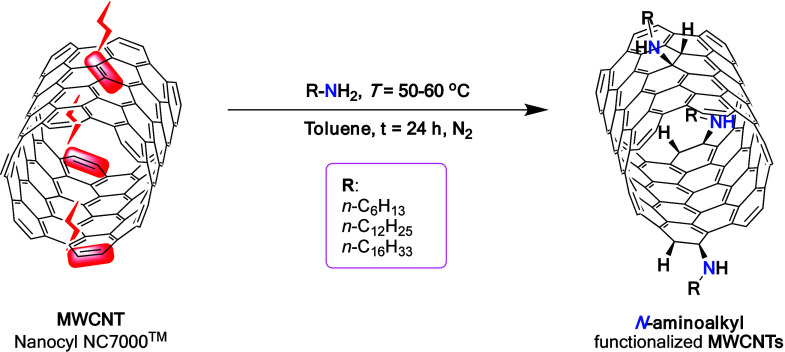
Scheme of chemical modification of MWCNTs with aliphatic amines, where *R* = *n*-C_6_H_13_, *n*-C_12_H_25_, and *n*-C_16_H_33_.

Functionalization of MWCNTs with terminal linear aliphatic amines yielded tailored nanomaterials engineered for use as high-performance lubricant additives. Although the exact mechanism of this reaction remains to be fully elucidated, it is postulated to involve a solvent-dependent electron transfer from the amine to the sp^2^-hybridized nanocarbon framework, followed by either radical coupling leading to a zwitterionic intermediate or proton transfer within the radical ion pair, ultimately generating neutral free radicals, which recombine and yield the final product (Figure S1).^31–34^ This strategy achieves atom economy, avoids side products and problematic waste, and operates efficiently under mild conditions. It thus provides a valuable alternative to diazotization/dediazotization protocols,^35,36^ despite recent advances in the selectivity of transformations exploring *n*-alkylamines.^37^

Furthermore, potential changes in the morphology of the CNT nanomaterial after the chemical modifications were tracked using cryo-TEM imaging (Figure 2).

**Figure 2:**
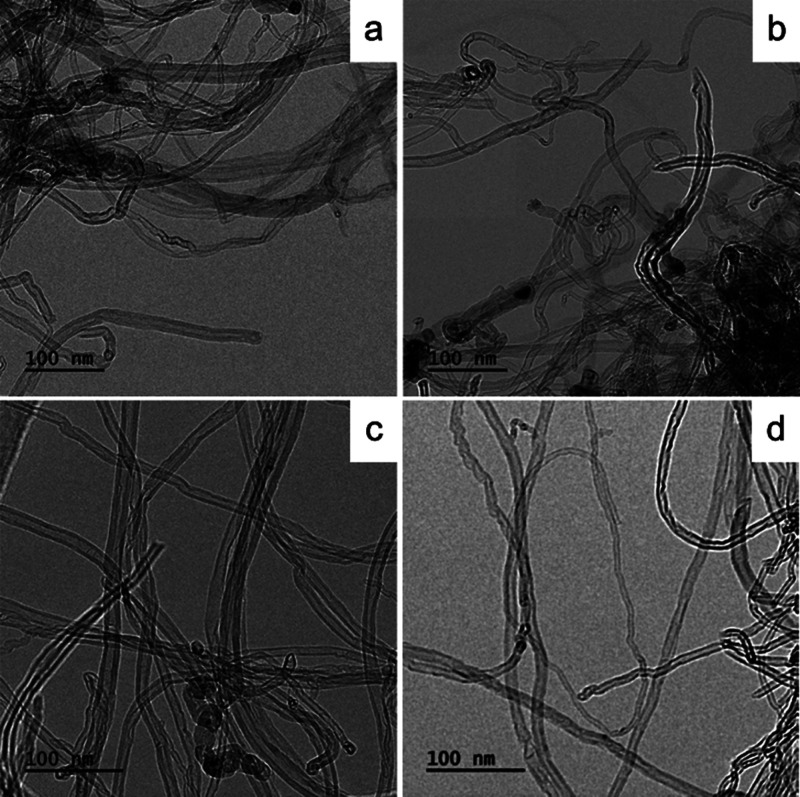
TEM images of (a) MWCNTs, (b) MWCNT-NH-C6, (c) MWCNT-NH-C12, and (d) MWCNT-NH-C16.

In the pristine MWCNTs (Figure 2a), a higher degree of entanglement and bundling was visible, along with the presence of some residual amorphous carbon or catalyst particles. Across all of the samples, the multiwalled, variously imperfect (kinks, corrugation, necking, encapsulation of residual catalyst particles, grooves, waviness, etc.) tubular *quasi*-1D individuals of the outer diameter in the ranges from 8 to 25 nm were clearly visible. The imaging indicated that functionalization (Figure 2b–d) did not significantly alter the overall morphology or structural integrity of the nanotubes. Nevertheless, after functionalization, the edges of the tubes were visible as fuzzier and one might observe more contrast along with glow, which could indirectly confirm functionalization of the MWCNT surface. Here, morphological changes served as supporting rather than primary evidence of functionalization, yet they clearly demonstrated the structural integrity of the nanotube framework postmodification—an outcome that is not guaranteed, as partial exfoliation into nanoribbons can occur under certain conditions.

Figure 3 presents thermogravimetric profiles acquired under pyrolytic conditions (N_2_ atmosphere), which substantiate the covalent *N*-alkylamino functionalization of MWCNTs. The data are exemplified by the MWCNT-NH-C6 derivative (Figure 3a), shown alongside the corresponding reference amine. Comparable thermograms were observed for the remaining two derivatives (Figure 3b).

**Figure 3:**
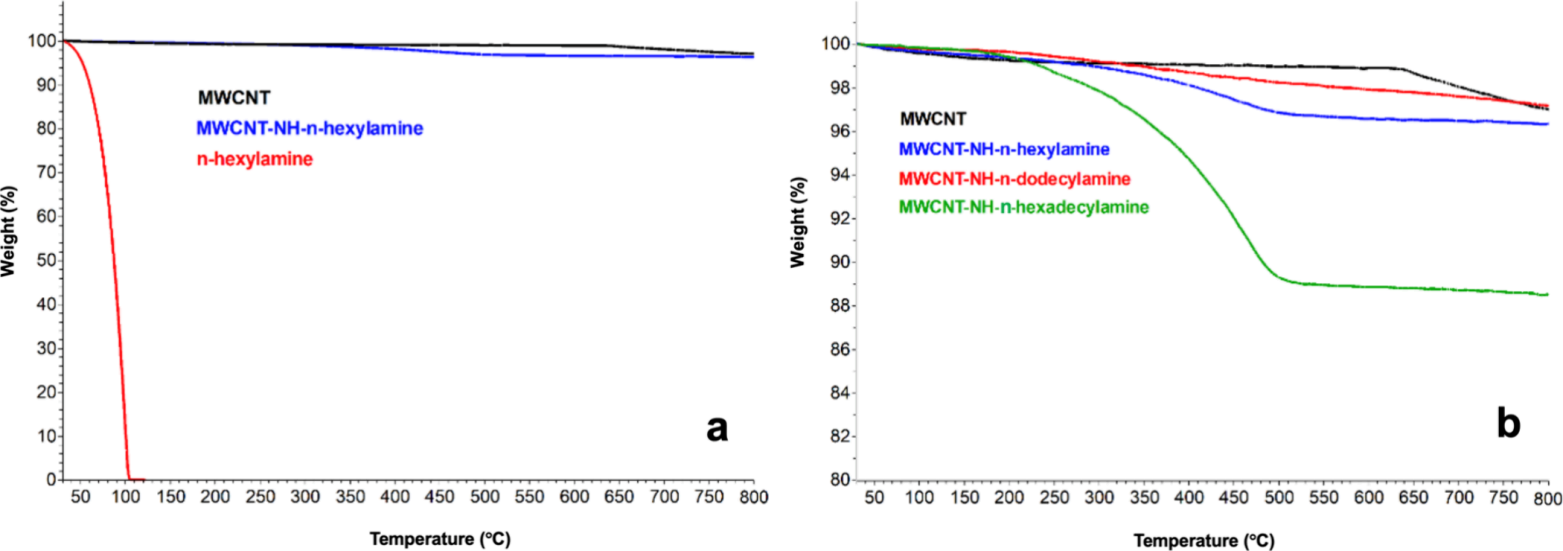
TGA curves of (a) pristine MWCNTs (black), pure *n*-hexylamine (red), and *N*-alkylamino-functionalized sample MWCNT-NH-C6 (blue) and (b) pristine MWCNTs (black) and *N-*alkylamino-functionalized MWCNTs: MWCNT-NH-C6 (blue), MWCNT-NH-C12 (red), and MWCNT-NH-C16 (green).

In order to determine the content of “to-nanotube-tethered” amine moieties, the differences in weight loss during heating of the unmodified versus modified MWCNTs in the temperature range from ∼120 to ∼680 °C were determined.^38^ The nanotube derivatives contained 2.8, 3.2, and 10.3 wt % of C12, C6, and C16 dangling *N*-alkylamino moieties, respectively.^39,40^ Overall, as revealed by the DTG curves (Figure S2), the temperatures corresponding to the maximum desorption rates of the functional groups—420 °C for C_6_- and 450 °C for C_16_-functionalized MWCNTs—strongly support the covalent nature of the surface modification. This is especially evident when contrasted with pure *n*-hexylamine, which exhibits complete evaporation well below 100 °C, with its boiling point of 131.5 °C. Additionally, this result indicated that the functionalization levels were nonmonotonic with respect to the molecular weight of the amines, likely due to variations in the conformational stability and affinity of the various alkyl chain length amines to the MWCNT surface. For example, intermediate alkyl chain lengths (C_12_) could tend to assemble into highly ordered, crystalline interfacial layers, which translates into the lowest functionalization degree.^41^ In contrast, longer chains (C_16_) typically form less-ordered, amorphous structures, resulting in higher loading, corroborating also a higher molecular weight of the modifying linker. On the other hand, the shorter C_6_ chains do not provide adequate anchoring to effectively stabilize the surface; however, their higher molecular mobility and reduced tendency to self-crystallize may enable somewhat greater adsorption onto the MWCNT surface compared to the C_12_ chains.^42^

Raman spectroscopy was employed to additionally elucidate the mode of MWCNT functionalization (Figure 4). A significant increase in the I_D_/I_G_ ratio, i.e., from 2.54 to 2.84, clearly demonstrated an increase in the number of *C*-sp^3^ atoms for the *N*-alkylamino MWCNTs. Such a behavior would be in line with the previously reported addition of amines^33^ and amino acids^31^ via the amine group to fullerene. The deconvolution presented herein shows a decrease in the G-band intensity, indicating a reaction involving sp^2^-bonding. This outcome suggests that amine groups were introduced at the sites of double bonds, leading to a reduction in the G-band signal and a concurrent increase in both the main and secondary D-bands. The functionalized MWCNTs also exhibited a higher G_v_-value, which supported the occurrence of covalent functionalization. Additionally, the higher G_l_-values observed in pristine MWCNTs—attributed to tube-end vibrations—suggest that end-cap opening may have occurred as a result of the functionalization process. Especially visible is a change in shape for the G peak, which presents crucial evidence for functionalization in the case of Raman spectroscopy. Furthermore, according to Rebelo et al.,^26^ MWCNTs exhibit a characteristic I_D_/I_G_ ratio due to their synthesis-derived high defectiveness.

**Figure 4:**
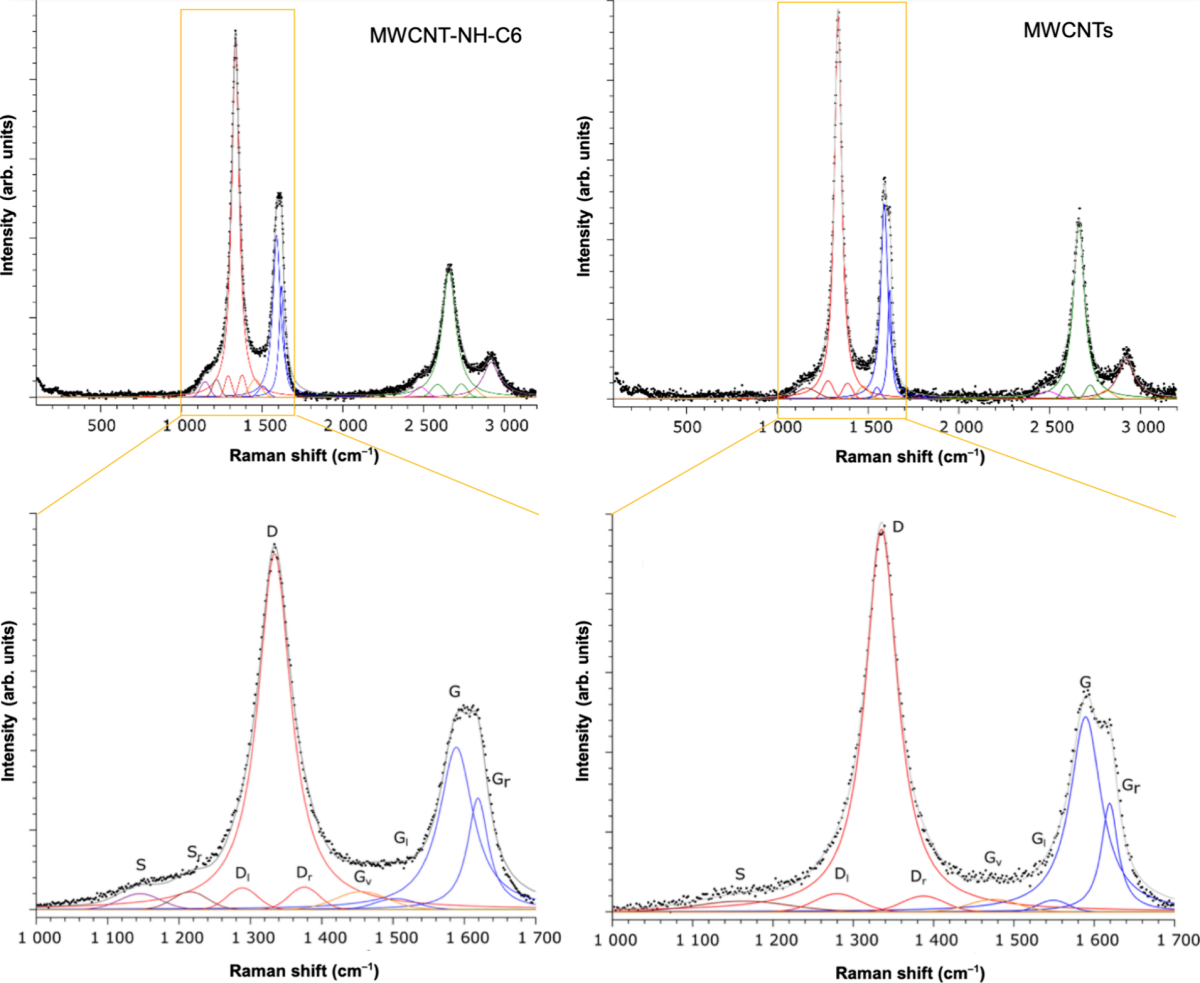
Raman spectra of the exemplary *N*-hexylamino MWCNTs (MWCNT-NH-C6) (left panel) versus pristine MWCNTs (right panel) with the magnified D- and G-regions (lower panel).

Moreover, Dresselhaus et al.^43^ highlighted the broader peak overlap observed in Raman spectra, which was attributed to the multiwalled structure of CNTs. Under excitation, this structure induces oscillations in each layer, leading to interactions among the layers. Additionally, long alkyl chains attached to the surface could cause entanglement of individual CNTs, which interferes with spectroscopic measurements and contributes to an increase in the G*_v_* component.

FTIR spectroscopy was also applied to confirm the covalent functionalization of MWCNTs, exemplarily with *n*-hexylamine. The comparative spectra of pristine MWCNTs, *n*-hexylamine, and MWCNT-NH-*n*-hexylamine are presented in Figure S3. The pristine MWCNTs exhibited characteristic bands near 3430 cm^–1^, attributable to ν­(O–H) stretching vibrations from the surface-adsorbed water or trace dangling hydroxyl groups.^44^ The weak absorption observed at ∼1620 cm^–1^ corresponds to the aromatic ν­(C=C) stretching mode of the *C*-sp^2^-hybridized framework.^45^ Additional minor features in the 1165–1050 cm^–1^ region could be assigned to δ­(C–O) bending, suggesting limited surface oxidation, consistent with prior literature.^46^ In contrast, the FTIR spectrum of *n*-hexylamine shows distinct ν_as_(CH_2_) and ν_s_(CH_3_) stretching vibrations at 2926 and 2854 cm^–1^, respectively, indicative of the aliphatic chain.^47^ A broad absorption at ∼3370 cm^–1^ is assigned to the asymmetric ν­(NH_2_) stretch of the primary amines.^48^ The bending mode δ­(NH_2_) appears near 1600 cm^–1^, while the δ­(CH_2_) and δ­(CH_3_) scissoring and wagging modes are observed at 1465 and 1375 cm^–1^. The band at ∼1020–1080 cm^–1^ is characteristic of ν­(C–N) stretching from the aliphatic amine group.^49^ With marginally different intensities and fingerprint regions, the other aliphatic amines display similar characteristics (Figure S4). In turn, the spectrum of MWCNT-NH-C6 displays a marked change. The presence of prominent methylene stretching bands at 2926 and 2854 cm^–1^ could confirm the successful grafting of the alkyl chain. The disappearance of the free amine ν­(NH_2_) stretch at ∼3370 cm^–1^, along with the emergence of a new band at ∼1570–1650 cm^–1^—attributed to δ­(NH) modes of secondary amide—supports covalent attachment via nucleophilic addition. Furthermore, the band at ∼1050–1100 cm^–1^, attributed to ν­(C–N) stretching, provides additional confirmation of the C–N bond formation. Overall, while FTIR offers valuable information, it is not the most conclusive method for characterizing surface modifications of CNTs, largely because their strong intrinsic absorption and scattering in the IR range^50^ tend to obscure characteristic signals, an issue that becomes especially pronounced at the low functionalization levels reported in this study.

XPS was employed to investigate the surface elemental composition of pristine MWCNTs and functionalized MWCNT-NH-C16, with the results summarized in Table S2. The atomic concentrations were calculated based on integrated peak areas corrected using the appropriate relative sensitivity factors (RSFs) as listed in Table S3. For pristine MWCNTs, the spectrum was dominated by the total carbon (*C*) signal (95.94%), with a minor total oxygen (*O*) content (4.06%) and no detectable N 1s signal, consistent with a largely unfunctionalized graphitic surface and trace oxygenated defects. In contrast, the MWCNT-NH-C16 sample exhibited a detectable nitrogen signal (0.34% area), marking it as the only sample in which nitrogen content exceeded the detection threshold (Figure S5). This observation supports evidence of the successful covalent attachment of *N*-alkylamine moieties. The presence of nitrogen, although low in concentration, is chemically significant and corroborates the thermogravimetric data (Figure 3 and Figure S2). Collectively, the XPS, FTIR, and Raman spectroscopy, as well as TGA/DTG results, confirm the effective chemical modification of MWCNTs with the *N*-alkylamine groups.

### Spectroscopic Evaluation of Preparation Stability by Measuring Transmittance Changes over Time

Table 1 shows the results from the stability measurement of the nanotube dispersions during storage expressed as TSI versus time. Briefly, the higher the TSI value, the more agglomeration-based changes that occur in the sample. Hence, a high TSI value indicates that the sample is unstable over time and *vice versa*.

**Table 1:** Stability of Nanotube Dispersions in the SN 650 Base Oil upon Storage, Expressed as TSI

*N*-alkylamino-functionalized MWCNTs	concentration in SN 650 [wt %]	TSI
MWCNT	0.001	1.9
	**0.01**	**0.4**
	0.1	0.3
	1	0.4
MWCNT-NH-C6	0.001	1.9
	**0.01**	**0.3**
	0.1	0.2
	1	0.2
MWCNT-NH-C12	0.001	4.2
	**0.01**	**0.3**
	0.1	0.3
	1	0.3
MWCNT-NH-C16	0.001	2.7
	**0.01**	**0.3**
	0.1	0.3
	1	0.3

Figure 5 shows the dependence of transmittance (*T*) on the height of the upper section of the test sample obtained under static conditions for a 7-day test for modified and unmodified MWCNT dispersions at 0.01 wt %, which was close to the concentration in the target greases, prepared as shown in Table 1 in the base hydrocarbon oil.

**Figure 5:**
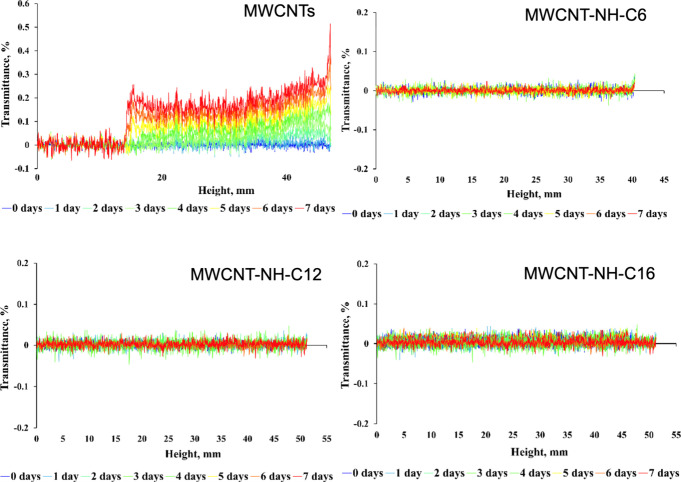
Transmittance changes as a function of the sample height of the various MWCNT dispersions at 25 °C.

First, the experiment showed that the upper region of the sample has become more transparent, confirming sedimentation of the aggregated nonfunctionalized MWCNTs. In turn, in the case of the all *N*-alkylamino MWCNTs, no significant transmittance changes were observed during storage for 7 days. Hence, the transmittance change profile confirmed the stability of the dispersion of modified MWCNTs within the studied period of time, while the fluctuations of the transmittance were not higher than 2%. The slightly higher deviations from the zero-transmittance were observed for the NH-C12- and NH-C16-modified MWCNTs, which could contribute to the larger agglomerates than for the NH-C6 sample. The calculated *TSI* dependence on time (Figure 6) showed that in the case of nonfunctionalized MWCNTs, oil dispersions displayed the highest TSI value (up to 1.9 within 7 days) at the lowest concentration.

**Figure 6:**
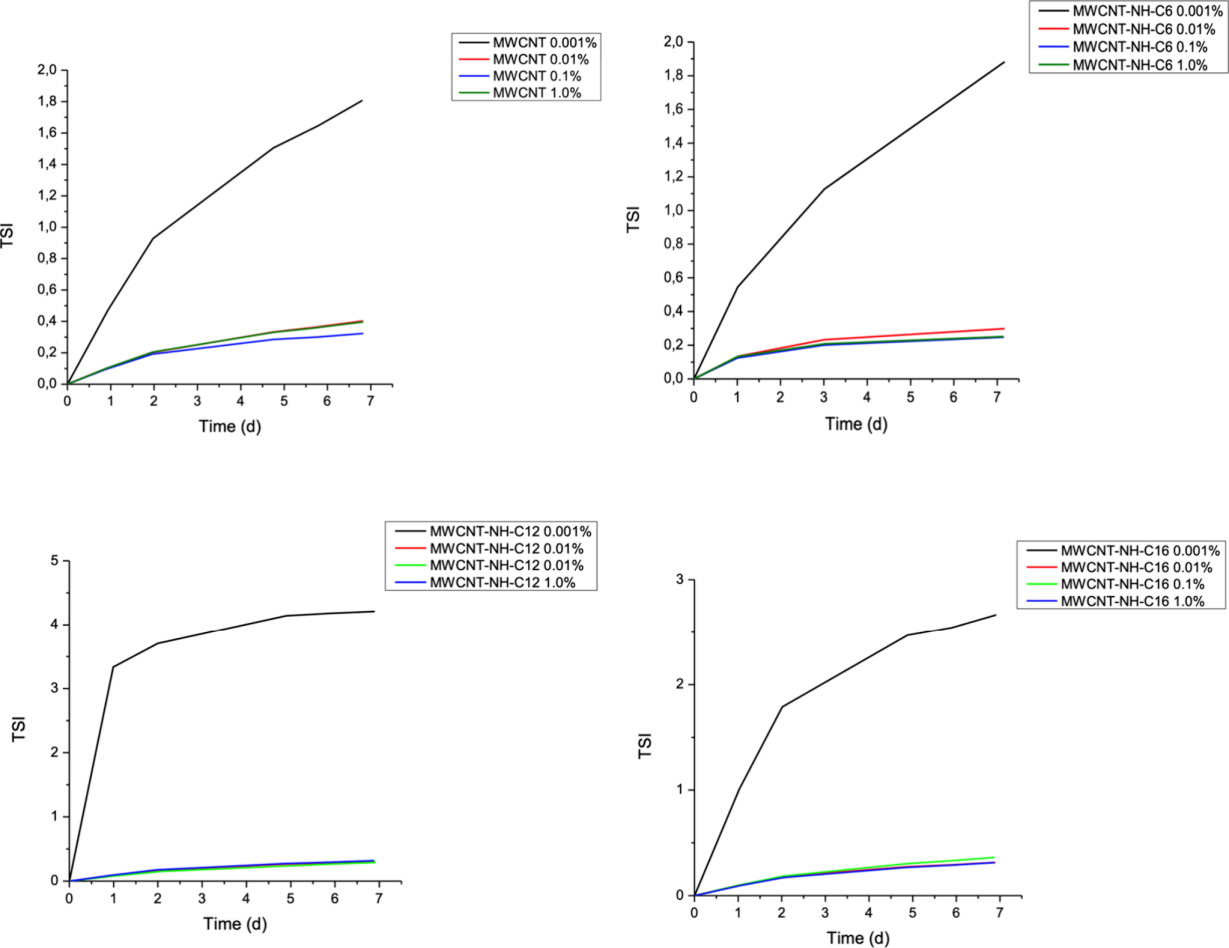
Time-dependent stability index (TSI) profiles of pristine and functionalized MWCNT dispersions at 25 °C; curves are provided solely as visual guides.

The increase in TSI was also the most pronounced among all nanotube dispersions studied. This trend can be attributed to the combined effects of nanotube individualization following functionalization and the concomitant rise in dispersion viscosity hampering Brownian motions.

### *N*-Alkylamino Functionalized CNTs as aDditive Components Lubricants for Highly Loaded Tribo-Pairs

Plastic greases, including the reference ones, were prepared according to the compositions (1–4) in Table 2.

**Table 2:** Composition of the Plastic Greases (1–4), in the Background of the Commercial Grease (Reference Grease) and the “Non-Nanotube-Enhanced” Grease (Reference grease_v0)

**component**	**reference grease**	**reference grease_v0**	**grease 1**	**grease 2**	**grease 3**	**grease 4**
base grease 1% thickener			98.73	98.73		
base grease 4% thickener	98.5	98.75			98.73	98.73
mixture of primary and secondary zinc dialkyldithiophosphates	1.5	0.50	0.50	0.50	0.50	0.50
nanographite		0.25	0.25	0.25	0.25	0.25
molybdenum disulfide		0.50	0.50	0.50	0.50	0.50
MWCNT-NH-C6			0.02			
MWCNT-NH-C12				0.02		0.02
MWCNT-NH-C16					0.02	
consistency class NLGI	2	2	2	2	2	2

Commercially available plastic lubricants often fall short of delivering optimal performance under severe loading conditions, particularly in heavily loaded tribological contacts.^51,52^ Operational issues frequently arise due to insufficient friction reduction and the accelerated wear of machine components. In practice, the performance of such plastic lubricants is typically evaluated using key tribological parameters, including weld load (ASTM D2596), oscillatory friction coefficient (SRV), sliding friction coefficient (μ), and mean wear scar diameter (ASTM D2266).^53^ But notably, there appear to be no reported plastic lubricants that simultaneously combine a wear scar diameter below 0.55 mm, an *SRV* friction coefficient below 0.65, a weld load exceeding 340 kG, and an NLGI consistency class of ≤2.^54,55^ This highlights a clear technological gap and the potential for novel nanoadditive-modified greases to significantly advance tribological performance beyond the current state of the art.

The basic tribological parameters for the new greases were compared to a commercially available grease from the same lube group, that is, obtained from the same base oil, using the same thickener and with the same NGLI consistency class, i.e., 2. Additionally, the “Reference grease_v0”, i.e., the grease noncontaining the nanotubes, was studied similarly. The three basic tribological parameters (according to the standards and five independent measurements; Figure 7) revealed the following characteristics.

**Figure 7:**
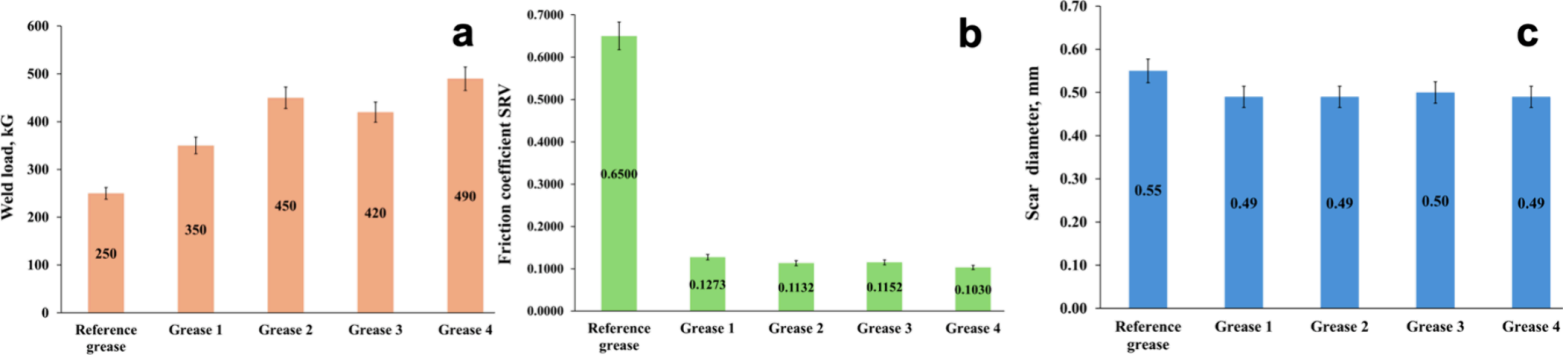
Determination of the three basic tribological parameters for the new plastic greases (1–4) at 25 °C: weld load (a), friction coefficient (b), and scar diameter (c).

First, all of the new plastic greases withstood the weld load in the range of 350–490 kG, with the highest value recorded to MWCNT-NH-C12. In comparison, typical commercial plastic greases, such as mixtures of the base oil (mineral oil, synthetic oil, methylsilicone oil, ester oil, or mixtures thereof), metal (Li, Ca, Mg, or Al) stearates, poly­(tetrafluoroethylene) (PTFE), and/or colloidal flame silica, displayed weld load values in the range of from 500 to 620 kG, while such a system still did not make it possible to reduce the value of the average scar diameter and the SRV oscillatory friction coefficient.^56^ Here, the reference grease_v0 exhibited a weld load of 325 kG, a wear scar diameter of 0.53 mm, and an SRV coefficient of friction of 0.1447, performance metrics predominantly influenced by the synergistic action of nanographite and nano-MoS_2_ additives (Figure S6). Hence, undoubtedly, the nanotube additives had a strong positive effect on lubrication by improving the properties of the oil film, leading to a reduction in friction through the formation of a protective film. The nanotubes could also induce the repair mechanisms at the tribo-pair, which affected the structure of friction surfaces through the effect of filling in irregularities and polishing. The reference grease showed an SRV friction coefficient of 0.65, reflecting a moderate level of friction when interacting with the tribo-pairs. This value means that the material has decent adhesion but is not too slippery or too rough. Such a coefficient of friction is often desirable in applications where a balance between friction and wear is required, such as in plain bearings or machine components operating under harsh conditions, but it is not suitable for a system operating under a high load, since at higher loads on the system, dry friction will occur, resulting in wear of mechanical components, leading to complete seizure.

The SRV-measured friction coefficients for the developed lubricants containing chemical MWCNTs as antiwear and antiseize additives were found to range between 0.1030 and 0.1273, with the lowest value observed, again, for the formulation enriched with the MWCNT-NH-C12 nanoadditive, designated as “grease 4”. Overall, these values correspond to an exceptionally low frictional response, indicative of highly lubricious surfaces. In practical terms, lubricants exhibiting such low coefficients of friction are highly advantageous in applications where minimizing frictional losses is critical—including heavily loaded tribo-pairs such as rolling element bearings, dynamic seals, high-precision mechanical assemblies, and energy-efficient actuation systems. Additionally, they hold promise for use in advanced sectors like aerospace mechanisms, electric motor components, and microelectromechanical systems (MEMSs), where reduced friction directly translates into improved reliability, energy efficiency, and component lifespan.

The third critical parameter—wear scar diameter, determined under standardized test conditions—serves as an indicator of lubricant quality and its suitability for demanding applications. This metric is especially vital when assessing lubricants for use in heavily loaded frictional interfaces where high contact stresses and severe wear can rapidly degrade materials. In such tribological systems, lubricants must exhibit exceptional wear resistance and sustain an effective lubricating film to reduce friction and avert catastrophic failure. The commercial reference lubricant yielded a scar diameter of 0.55 mm, reflecting moderate surface wear under the test conditions. In contrast, the newly formulated greases incorporating *N*-alkylamino MWCNTs produced smaller scar diameters, ranging from 0.49 to 0.50 mm—practically independent of the nanoadditive variants—demonstrating superior antiwear performance. Representative micrographs used to determine wear scar diameters, among the three measurements on three balls (nine (9) independent measurements for each grease), are demonstrated in Figure 8.

**Figure 8:**
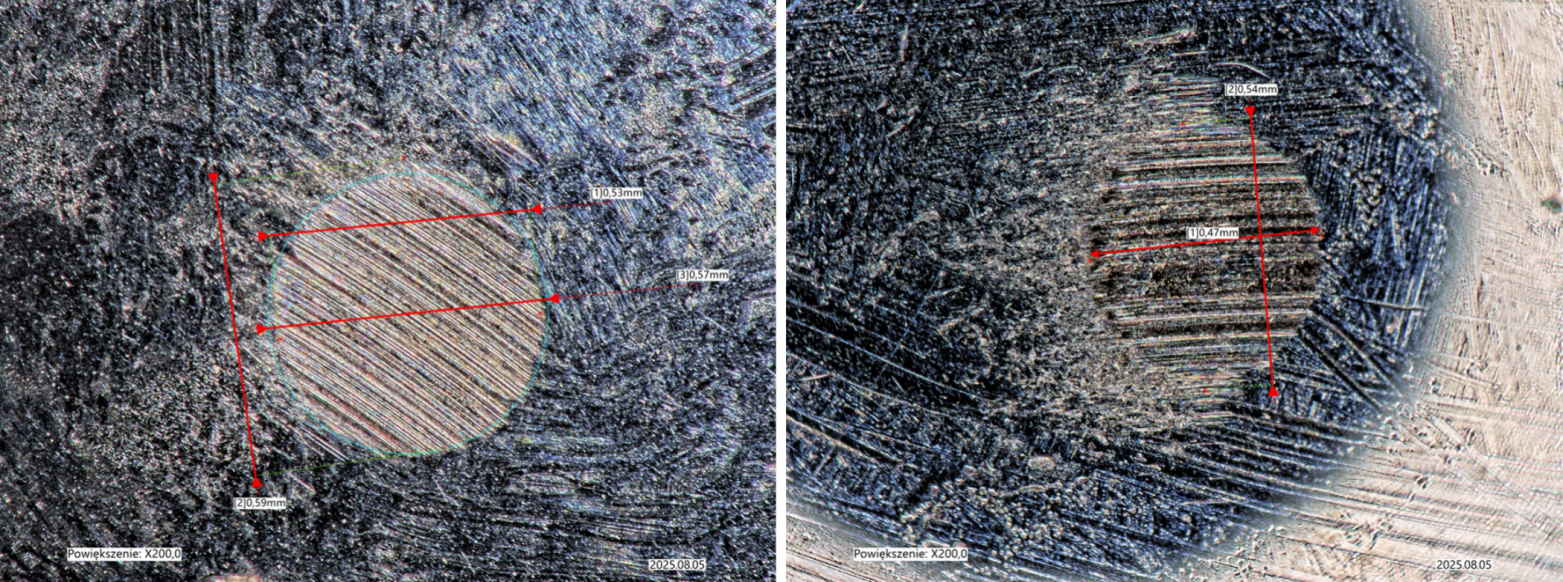
Representative micrographs used to determine wear scar diameters for the Reference grease_v0 (left) and “grease 1” containing 0.02 wt % of MWCNT-NH-C6 (right).

This improvement suggests that *N*-alkylamino-MWCNT-enriched lubricants provide enhanced surface protection against wear and scuffing, likely due to mechanisms such as tribofilm formation, load-bearing capacity enhancement, and micropolishing effects.

Generally, the tribological performance of various grease- and oil-based lubricants augmented with nanoparticles has been extensively investigated, yet a direct comparison remains inherently complex due to variations in test protocols, applied loads, durations, temperatures, and base formulations. The results achieved in this study, contextualized against representative state-of-the-art solutions, are summarized in Table 3. Clearly, many of these systems either rely on high nanoparticle loadings, exhibit limited reproducibility, or require chemically elaborate and cost-intensive synthesis routes. These results underscore the efficacy of herein presented functionalized MWCNTs as robust friction-reducing and antiwear additives, offering scalable and compositionally simpler alternatives to existing nanoparticle-enhanced lubricants. Despite the inherent variability in test methodologies, the observed improvements across key metrics—friction, wear, and extreme pressure resistance—affirm the superior tribological performance of our materials and their potential for industrial deployment in high-load and high-durability applications.

**Table 3:** Comparative Tribological Performance of Reported Greases Enhanced with Nanomaterials in the Background of Present MWCNT-Based Grease Formulations^a^

MWCNT grease	*c*_MWCNT_	tribo-test	load	*T*, *t*	CoF, –	WSD, mm	WL, kG	ref.
Ca^2+^ grease	0 wt %	four-ball	40 kG	75 °C, 1 h	0.12	0.75	200	^57^
	3 wt %				0.06	0.51	276	
mineral gear oil	0.0 wt %	ASTM D4172	40 kG	75 °C, 1 h	0.842	3.624	200	^58^
	0.1 wt %				0.440	2.431	200	
	0.5 wt %				0.210	2.225	200	
	0.6 wt %				0.411	2.313	200	
Li^+^/Ca^2+^/Al_2_O_3_ grease	0 wt %	four-ball	40 kG	75 °C, −	0.014	0.22	260	^59^
	4 wt %				0.010	0.15	280	
PAO oil + MoS_2_ (1.0 wt %)	0.0 wt %	ball-on-disc	20 N	RT, 1 h	0.12	0.321		^60^
	7.5 wt %				0.13	0.231		
ionic liquid/Cu NPs	0.1 wt %	block-on-ring	‘Low’	RT, –	<0.01			^61^
SAE 5W-30 base oil	0.00 wt %	ASTM D4172 + EP	40 kG	75 °C, 1 h			200	^62^
	0.06 wt %				0.040	0.80	250	
mineral plastic grease		SRV + four-ball	40 kG	RT, 1 h	0.6500	0.55	250	This work
v0 = (mineral plastic grease+nanographite + MoS_2_)	0.00 wt %	SRV + four-ball	40 kG	RT, 1 h	0.1447	0.53	325	This work
v0 + MWCNT-NH-C6 = v1	0.02 wt %	SRV + four-ball	40 kG	RT, 1 h	0.1273	0.49	350	This work
v0 + MWCNT-NH-C12 = v2	0.02 wt %	SRV + four-ball	40 kG	RT, 1 h	0.1132	0.49	450	This work
v0 + MWCNT-NH-C16 = v3	0.02 wt %	SRV + four-ball	40 kG	RT, 1 h	0.1152	0.50	420	This work
v0 + MWCNT-NH-C12 = v4	0.02 wt %	SRV + four-ball	40 kG	RT, 1 h	0.1030	0.49	490	This work

a WL – weld load; RT – room temperature.

Regarding the underlying tribological mechanisms, the formation of a multifunctional 3D tribofilm appears to be the most plausible explanation for the performance enhancement induced by *N*-alkylamino-functionalized MWCNTs. Short and thin MWCNTs have been previously demonstrated to act as nanoscale bridges, interconnecting other nanostructures—including similarly structured,^63^ longer,^64^ subzipped^65^ MWCNTs and graphene^66^—into robust 3D networks, akin to those formed by lithium-based soaps. An additional critical factor influencing the performance of MWCNT-based greases is their enhanced thermal conductivity,^67^ which facilitates efficient heat dissipation from tribological contact zones, thereby extending the service life of mechanical components by suppressing oxidative degradation and thermally induced wear mechanisms.

## Conclusions

This study establishes *N*-alkylamino-functionalized MWCNTs, synthesized via a one-step direct nucleophilic addition of *n*-alkylamines to commercially available MWCNTs, as an effective high-performance nanoadditive for advanced plastic lubricant formulations. At low additive concentrations, these functionalized nanomaterials deliver substantial tribological enhancements over state-of-the-art commercial greases while maintaining exceptional dispersion stability in base mineral oils across extended operational durations.

All four formulated greases demonstrated consistent and significant improvements in critical tribological metrics, including a reduced wear scar diameter, a stable oscillatory *SRV* CoF up to 0.10, and a weld load capacity reaching 490 kG, all within the desirable consistency of greases, i.e., class 2. These results underscore the dual effectiveness of the *N*-alkylamino MWCNTs in mitigating both friction and wear under high-load conditions—traits central to the demands of modern tribo-systems. Beyond performance, the additive strategy introduces practical manufacturing advantages. Dispersing nanomaterials directly into the base oil streamlines the formulation process, minimizes dust-related occupational hazards, and enables safer and more scalable production without compromising consistency.

Looking ahead, key research directions include (a) systematic investigations into the role of alkyl chain length and functional group density on tribofilm formation and durability, (b) extending compatibility studies to synthetic and biobased lubricants, (c) exploring synergistic effects with conventional extreme pressure and antiwear additives, and (d) evaluating performance at scale through long-term field trials in operational environments.

From an applied standpoint, the developed greases show high promise for deployment in heavy-duty bearings, gear systems operating under boundary or mixed lubrication regimes, and advanced mechanical platforms—including robotics, aerospace actuators, electric drivetrains, and high-speed precision spindles—where friction reduction and wear mitigation are pivotal to efficiency, reliability, and extended service life.

## Supplementary Material



## Notes

Scheme of preparation of MWCNT and *N*−aminoalkyl MWCNT dispersions in the base mineral oil with the corresponding photos, postulated mechanism of the nucleophilic addition of n-alkylamine (here exemplified by *n*-hexylamine) to CNT *C*-sp^2^-hybridized framework, as well as TGA and DTG curves, XPS spectra, FTIR spectra of pristine MWCNTs versus their *N*−aminoalkyl MWCNT analogs; time−dependent SRV coefficient of friction (CoF) curves for ‘Reference grease_v0’ (noncontaining nanotubes) and ‘grease 1’ enabling calculation of SRV CoFs (PDF)
