# Supplementary material for: N‑Alkylamino-Functionalized Multiwalled Carbon Nanotubes for Advanced Grease Applications: Stability and Tribological Enhancement
Source: ACS Omega. 2025 Oct 28;10(44):53224–37. doi: 10.1021/acsomega.5c07880 (PMC12613121; doi:10.1021/acsomega.5c07880)
Supplement: Supplementary file 1 [file ao5c07880_si_001.pdf]

SUPPLEMENTARY INFORMATION

for

N-Alkylamino Functionalized Multi-walled Carbon Nanotubes for Advanced Grease Applications: Stability and Tribological Enhancement

Ilona Scudło[1,2], Julia Woch[2], Szymon Ruczka[1], Kamil Korasiak[2],

Ewa Sabura[2], Katarzyna Gębura[2], Agata Blacha-Grzechnik[3,4], Sławomir Boncel[1,4,5,*]

[1]*Silesian University of Technology, Faculty of Chemistry, Department of Organic Chemistry, Bioorganic Chemistry and Biotechnology, NanoCarbon Group, 4 Bolesława Krzywoustego Street, 44-100 Gliwice, Poland*

[2]*Łukasiewicz Research Network – Institute of Heavy Organic Synthesis 'Blachownia', 9 Energetyków Street, 47-225 Kędzierzyn-Koźle, Poland*

[3]*Silesian University of Technology, Faculty of Chemistry, Department of Physical Chemistry and Technology of Polymers, 9 Ks. Marcina Strzody Street, 44-100 Gliwice, Poland*

[4]*Silesian University of Technology, Centre for Organic and Nanohybrid Electronics (CONE), 22B Stanisława Konarskiego Street, 44-100 Gliwice, Poland*

[5]*NanoCarbon Group Ltd., 7 Ks. Marcina Strzody Street, 44-100 Gliwice, Poland*

*Corresponding author: slawomir.boncel@polsl.pl

Table S1 Scheme of preparation of MWCNT and *N*-aminoalkyl MWCNT dispersions in the base mineral oil.

**Stage**	**1**	**2**	**3**	**4**
	Suspension of MWCNTs and *N*-aminoalkyl MWCNTs in base oil – manual mixing	High speed homogenization	Ultrasonication	Dispersion of MWCNTs and *N*-aminoalkyl MWCNTs in base oil
Photo	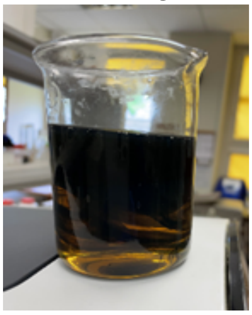	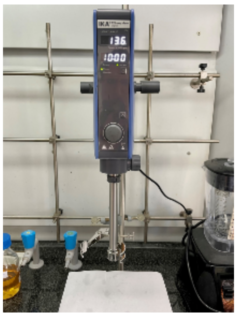	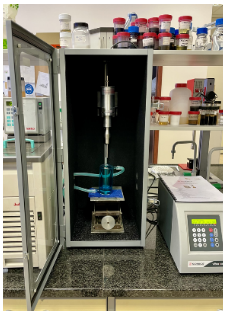	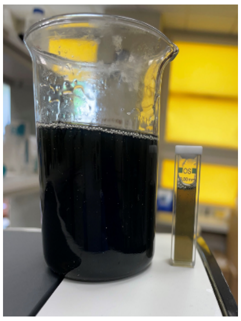
Remarks	Non-homogeneous suspension	Homogenizer T25 IKA 13000 rpm, 5 min	Sonicator SONICS VC 505	Homogeneous dispersion

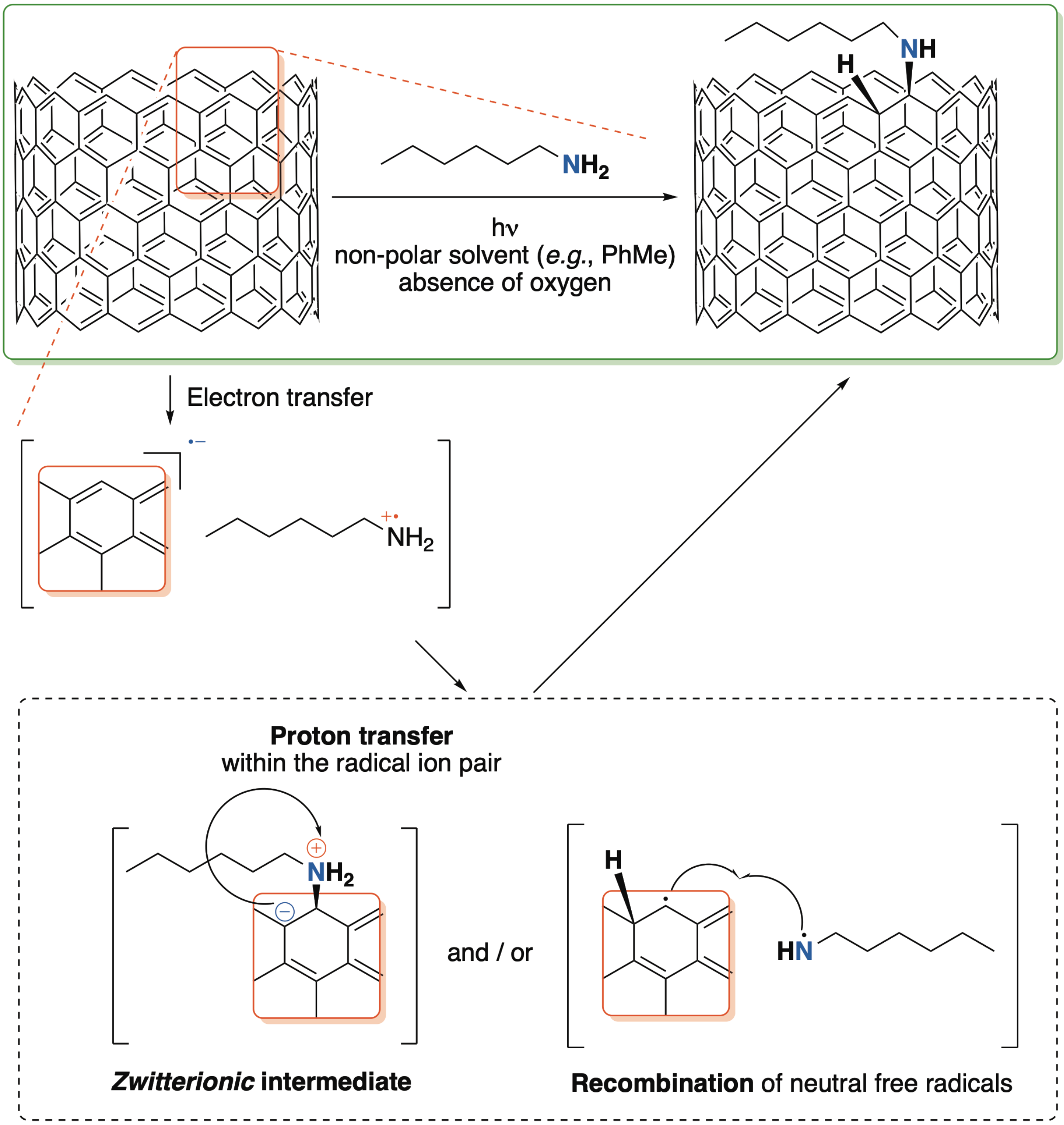

Fig. S1 Postulated mechanism of the nucleophilic addition of *n*-alkylamine (here exemplified by *n*-hexylamine) to carbon nanotube *C*-sp^2-hybridized framework. Current evidences suggest a solvent-dependent electron transfer from the amine donor to the CNT wall, and then, the resulting radical ion pair (*zwitterionic* intermediate) may undergo either radical coupling *via* a zwitterionic intermediate or proton transfer; ultimately, the sequence generates neutral radicals that recombine to deliver the final functionalized product – *N*-aminoalkyl functionalized MWCNTs.

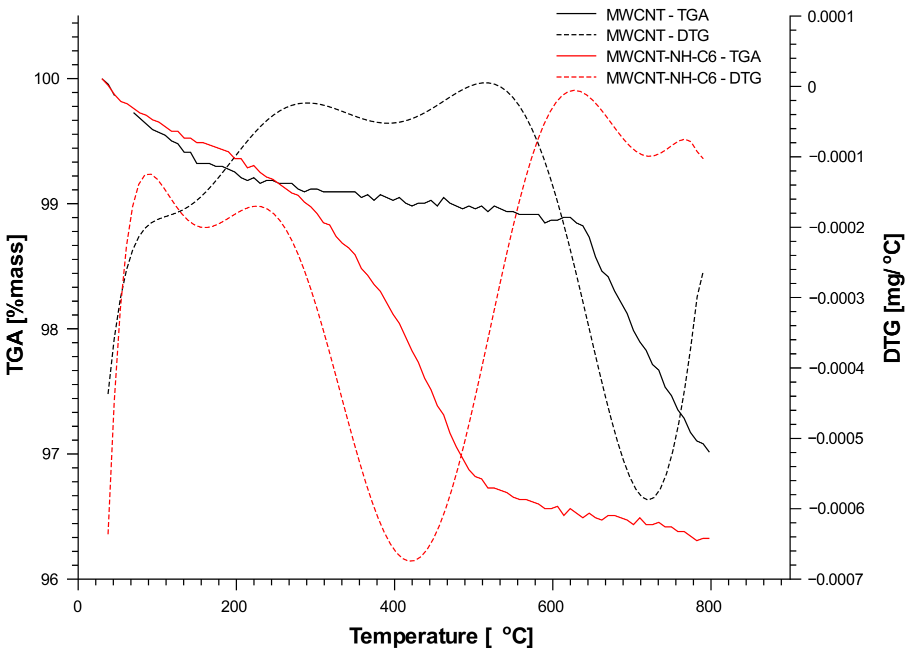

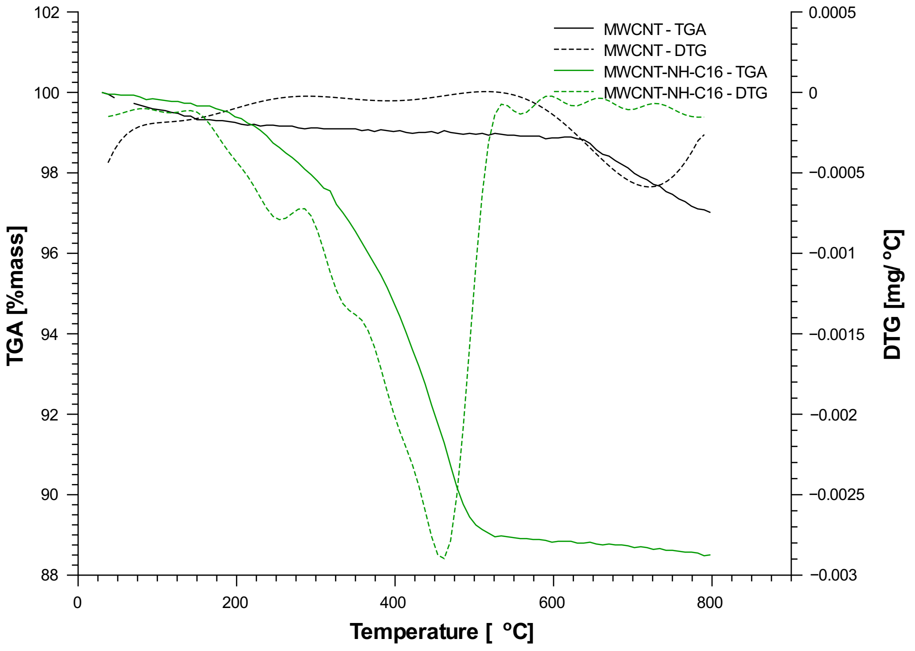

Fig. S2 Thermogravimetric analysis (TGA) and derivative thermogravimetry (DTG) curves of MWCNT-NH-C6 (*top*) and MWCNT-NH-C16 (*bottom*), shown in comparison with pristine MWCNTs.

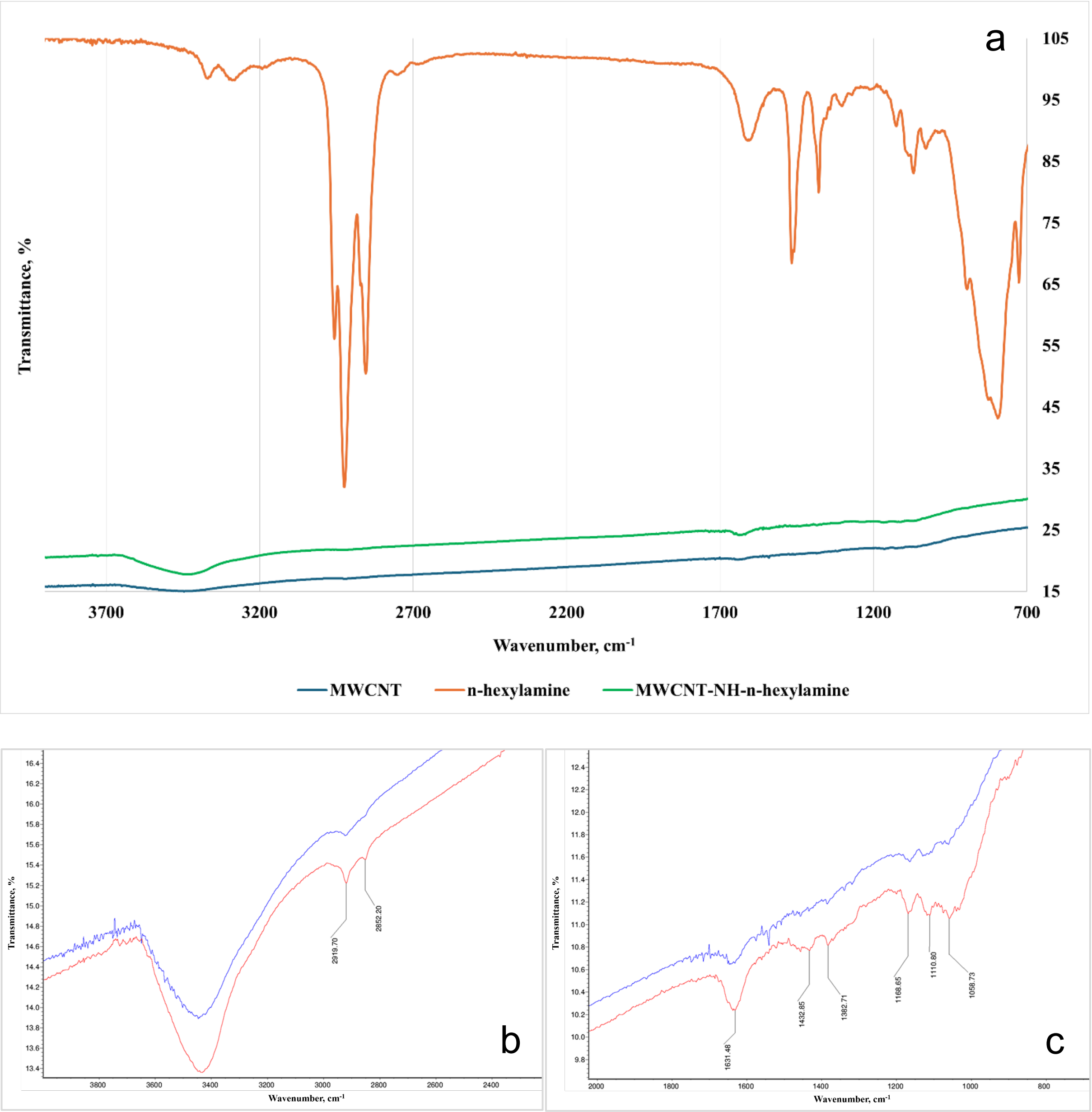

Fig. S3 FTIR spectra of unmodified MWCNTs, *n*-hexylamine, and MWCNTs modified by *n*-hexylamine (MWCNT-NH-C6) in the range of 3900–700 cm^{-1} (**a**) and the corresponding FTIR spectra of: unmodified MWCNTs (*blue*) and MWCNT-NH-C6 (*red*) in the range of: 4000–2200 cm^{-1} (**b**), 2000–800 cm^{-1} (**c**).

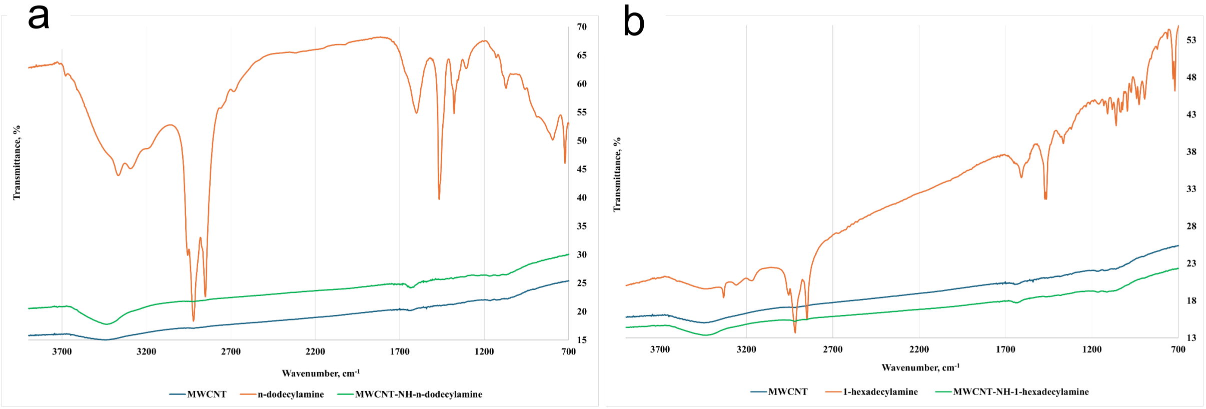

Fig. S4 FTIR spectra in the 3900–700 cm^{-1} range of: MWCNTs, *n*-dodecylamine, and MWCNT-NH-C12 (**a**) and MWCNTs, *n*-hexadecylamine, and MWCNT-NH-C16 hexadecylamine (**b**).

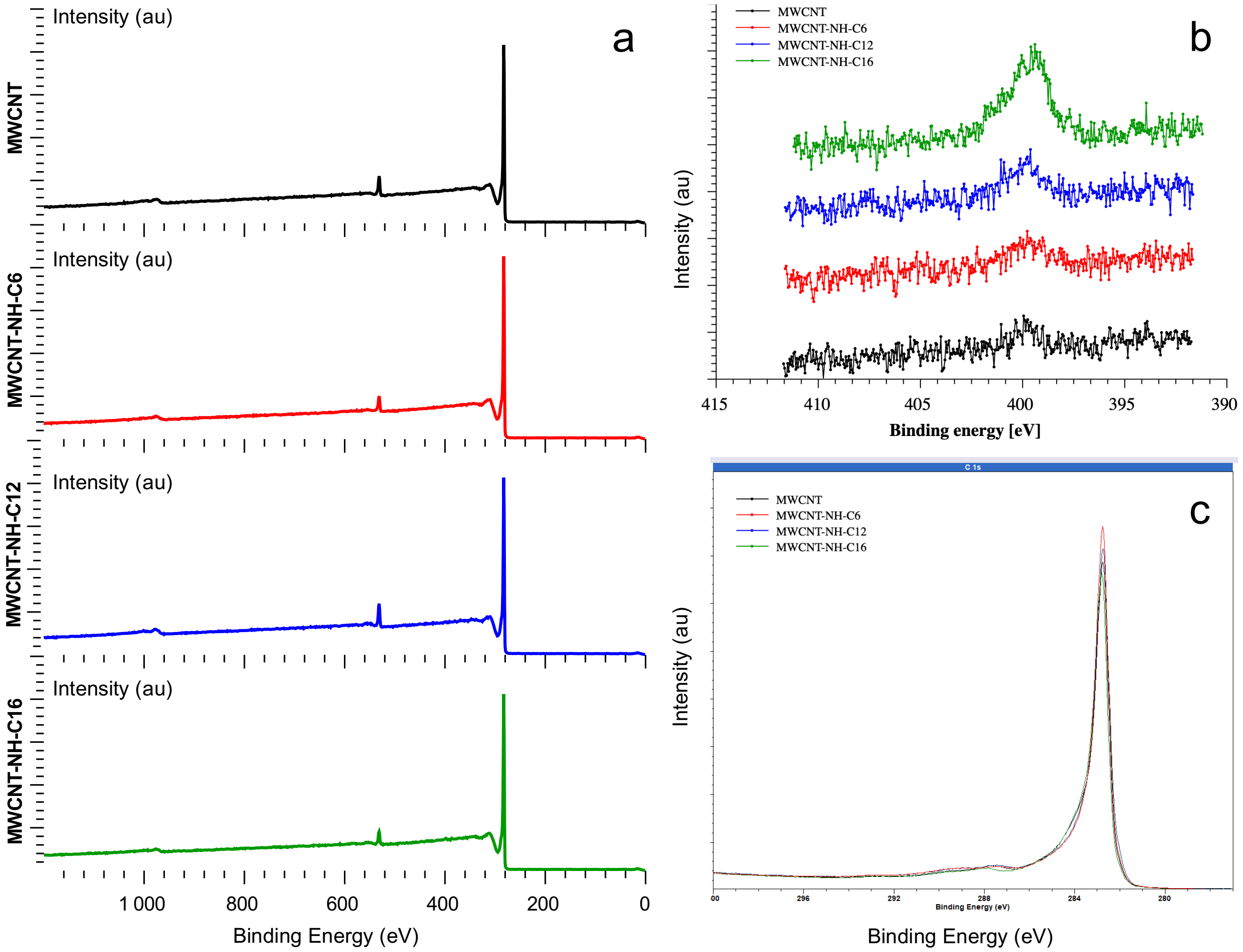

Fig. S5 XPS spectra of pristine MWCNTs and their *N*-aminoalkyl-functionalized analogues: (a) survey (wide) spectra; (b) high-resolution N 1s region; and (c) high-resolution C 1s region.

Table S2 Concentration (at. %) of *C*, *O*, *N* elements in the *f*-MWCNT samples

Sample	Total *C* conc. (at. %)	Total *O* conc. (at. %)	Total *N* conc. (at. %)
MWCNTs	95.94	4.06	0.00
MWCNT-NH-C16	96.76	2.89	0.34

Table S3 Relative Sensitivity Factor (RSF) values for the element lines used

Element line	RSF
C 1s	1.00
O 1s	2.93
N 1s	1.80

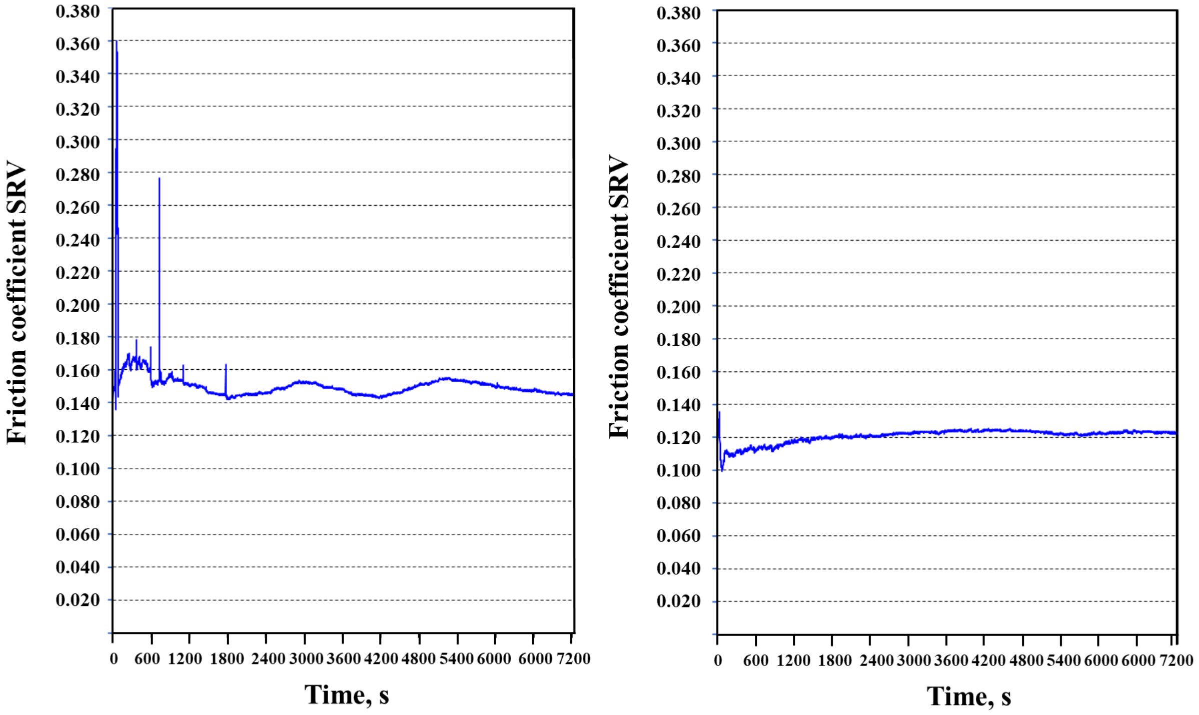

Fig. S6 Time-dependent *SRV* coefficient of friction (*CoF*) curves for ‘Reference grease_v0’ (non-containing nanotubes) (*left*) and ‘Grease 1’ (Base grease 1% thickener = 98.73 wt.%, Mixture of primary and secondary zinc dialkyldithiophosphates = 0.50 wt.%, Nanographite = 0.25 wt.%, Molybdenum disulfide = 0.50 wt.%; MWCNT-NH-C6 = 0.02 wt.%) (*right*) enabling calculation of *SRV CoF* as 0.1447 and 0.1273, respectively.
